# Occurrence of Double Bond in *π*-Aromatic Rings: An Easy Way to Design Doubly Aromatic Carbon-Metal Structures

**DOI:** 10.3390/molecules26237232

**Published:** 2021-11-29

**Authors:** Nikolay V. Tkachenko, Alvaro Muñoz-Castro, Alexander I. Boldyrev

**Affiliations:** 1 Department of Chemistry and Biochemistry, Utah State University, 0300 Old Main Hill, Logan, UT 84322-0300, USA; nikolay.tkachenko95@gmail.com; 2 Grupo de Química Inorgánica y Materiales Moleculares, Facultad de Ingeniería, Universidad Autonoma de Chile, El Llano Subercaseaux, Santiago 2801, Chile; alvaro.munoz@uautonoma.cl

**Keywords:** aromaticity, double-aromaticity, metallabenzynes, Hückel’s aromaticity rule

## Abstract

A chemical bonding of several metallabenzenes and metallabenzynes was studied via an adaptive natural density partitioning (AdNDP) algorithm and the induced magnetic field analysis. A unique chemical bonding pattern was discovered where the M=C (M: Os, Re) double bond coexists with the delocalized 6c-2e π-bonding elements responsible for aromatic properties of the investigated complexes. In opposition to the previous description where 8 delocalized π-electrons were reported in metallabenzenes and metallabenzynes, we showed that only six delocalized π-electrons are present in those molecules. Thus, there is no deviation from Hückel’s aromaticity rule for metallabenzynes/metallabenzenes complexes. Based on the discovered bonding pattern, we propose two thermodynamically stable novel molecules that possess not only π-delocalization but also retain six σ-delocalized electrons, rendering them as doubly aromatic species. As a result, our investigation gives a new direction for the search for carbon-metal doubly aromatic molecules.

## 1. Introduction

Since the pioneering work of Thorn and Hoffmann investigating the delocalization in metallocycles [1], and a consequent isolation of osmebenzene by Roper et al. [2], a huge variety of metalla-aromatic structures have been discovered experimentally and studied computationally [3,4,5,6,7,8,9]. Those species can be described as usual aromatic compounds where one or several carbon atoms are replaced with a transition metal atom. Such a peculiar combination of a transition metal and a carbon-framework can produce a variety of interesting chemical bonding patterns. The study of metallocycles is important not only for the broad understanding of chemical bonding concepts and applicability of the aromaticity term but also as useful for synthetic organic chemistry [10,11,12,13].

**Figure 1:**
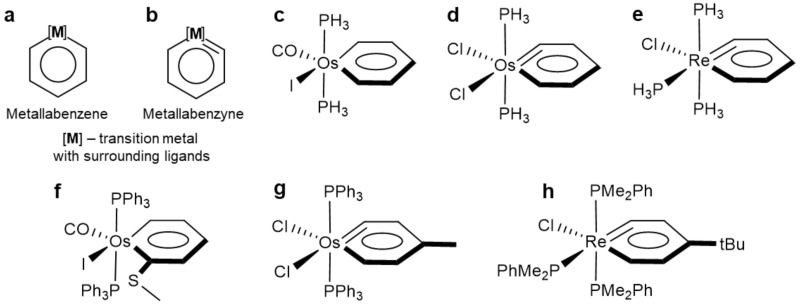
Structures of metallabenzenes and metallabenzynes. Structures (**a**) and (**b**) are general structures of metallabenzenes and metallabenzynes, respectively. Structures (**c**–**e**) are considered in this study and are model structures for previously synthesized complexes (**f**–**h**).

One very important subgroup of metalla-aromatics is metallabenzenes (Figure 1a), metallacycle analogs to benzene. Chemists have managed to synthesize a huge variety of these complexes, incorporating various metals into the aromatic ring such as: osmium [14,15,16,17,18,19,20,21,22,23], rhenium [24,25,26], platinum [27,28,29,30], and iridium [31,32,33,34,35,36,37]. It was shown that metallabenzenes bear an appreciable aromatic character which is reflected in rather high aromatic stabilization energies [38,39,40,41] and negative nucleus-independent chemical shift values [42,43,44,45]. In the work of Thorn and Hoffmann, authors suggested that the metallabenzenes should have six π electrons, two of which come from the metal atom (d_xz_ orbital), while the remaining 4 electrons come from the C_5_H_5_^−^ fragment [1]. Martin et al. showed a cognate conclusion, noting a striking similarity between orbitals of benzene and metallabenzenes [44]. Interestingly, more recent studies do not share this point of view, introducing different numbers of delocalized π-electrons. Thus, Schleyer proposed that the d_yz_ orbital is also participating in π-delocalization, and, thus, eight π-electrons are delocalized in the π-system [3]. This result contradicts with the Hückel [4n + 2] aromaticity rule; however, authors discussed that only two delocalized orbitals have the Hückel character, while another two orbitals (where the d_yz_ orbital participates in delocalization) have the Möbius character [46] for which Hückel’s rule does not work. Later, Fernandez and Frenking proposed their interpretation of metallabenzenes aromaticity, showing the presence of ten π-electrons, where *p*-orbitals of in-plane ligands also participate in the π-delocalization [38,47]. All alternative interpretations were based on the analysis of canonical molecular orbitals (MOs) and studied the degree of stabilization that comes from the metal *d*-orbitals. In this work, we are trying to address this issue with the AdNDP localization technique that can explicitly show the degree of delocalization within the metallacycle.

Another quite intriguing subgroup of the metalla-aromatic compound is metallabenzynes (Figure 1b). Those complexes are of particular interest since they combine the double M=C bond and π-conjugated aromatic ring. Such a peculiar chemical bonding regime can help design new molecules with unprecedented physical and chemical properties.

In this study, we analyze the chemical bonding patterns of various model metallabenzene and metallabenzyne complexes (Figure 1c–e) which are analogues of experimentally synthesized complexes (Figure 1f–h) [14,48,49]. From the AdNDP analysis, only six π-electrons were found to participate in the delocalization in both metallabenzenes and metallabenzynes, agreeing with the Thorn and Hoffmann description. Based on the chemical bonding analysis of osmabenzyne and rhenabenzyne, two novel structures that possess doubly aromatic six-membered carbon-metal ring were designed.

## 2. Theoretical Methods

All structures were initially preoptimized using a PBE0 [50] DFT functional and LANL2DZ [51] basis set. Subsequent refinement of structural data was conducted using a large def2-TZVP [52] basis set. A chemical bonding analysis was performed using the AdNDP [53,54] algorithm as implemented in AdNDP 2.0 code. The AdNDP method can represent a chemical bonding pattern in terms of both Lewis bonding elements (lone pairs (1c–2e) and 2c–2e bonds) as well as delocalized bonding elements (such as nc-2e (n  >  2) bonds), allowing one to describe highly delocalized electrons associated with the concept of aromaticity. This technique is widely used in describing chemical bonding patterns of various metal-containing clusters [55,56,57,58,59,60], aromatic 2D-materials [61,62,63], and doubly aromatic metal systems [64,65]. All AdNDP calculations were performed at the PBE0/def2-TZVP level of theory. It was shown before that the PBE0 functional could overestimate electron delocalization and aromaticity in planar aromatic systems [66]. To avoid this problem, we reoptimized geometries and performed the AdNDP analysis using the CAM-B3LYP functional [67] that does not suffer from a delocalization error. We note that the obtained AdNDP results reproduce results obtained with the PBE0 functional showing that the found chemical bonding pattern is DFT-functionally independent. The frequency calculations were performed using the harmonic approximation, and all reported structures were confirmed to be minimal due to the absence of imaginary frequencies. To assess the aromaticity with a quantitative parameter, NICS [68] calculations were performed at the PBE0/def2-TZVP level. The ChemCraft 1.8 software was used to visualize chemical bonding patterns and geometries of investigated compounds. The Gaussian 16 software was used for all quantum chemical calculations described above [69].

The nucleus-independent shielding tensors (σ_ij_) [70,71,72] were calculated within the GIAO formalism by using the ADF2019 code [73], employing the hybrid PBE0 [50] functional and an all-electron STO-TZ2P basis set, placed in a three-dimensional grid in order to evaluate the induced field (B^ind^), upon an external magnetic field (B^ext^), related via B_i_^ind^  =  −σ_ij_B_j_^ext^ [74,75,76]. For convenience, the i and j suffixes are related to the x-, y-, and z-axes of the molecule-fixed Cartesian system (i, j  =  x, y, z). The values of B^ind^ are given in ppm in relation to B^ext^. Relativistic effects were considered through the ZORA Hamiltonian [77].

## 3. Results and Discussion

### 3.1. Hückel’s [4n+2] π-Aromaticity of Metallabenzenes and Metallabenzynes

To model the synthesized rhenium and osmium complexes (Figure 1f–h), we replaced bulky Ph_3_P- and PhMe_2_P- groups with a PH_3_- ligand, while we preserve all atoms in the aromatic rings as they are in the experimental structure. Such a substitution does not affect chemical bonding in the metallocycle itself and will reflect the delocalization pattern properly. The optimized geometries reproduce the M-C (M: Os, Re) and C-C distances within the aromatic ring with a good precision (bond lengths’ deviations from the experimental data are within the 0.1–0.01 Å range). The alternation of C-C inside the ring is not large, and all bonds have the same length within the ~0.03 Å for Os structures and ~0.05 Å for the Rhenium complex. To ensure that the constructed single-determinant singlet wavefunction can be applied in those cases, we checked the stability of the wave function (WF) showing the absence of any RKS to UKS instabilities. We also performed the optimization of the complexes in the triplet open-shell configuration and found the triplet states are appreciably higher in energy than the singlet states. Those facts show that the DFT approximation is valid in this case, and the singlet single-determinant WF could be used to describe the systems considered. For the sake of simplicity, we introduce the following notation for the investigated complexes: [Os(CO)I(PH_3_)_2_(C_5_H_5_)]-[**Os**]C_5_H_5_, [OsCl_2_(PH_3_)_2_(C_5_H_4_)]-[**Os**]C_5_H_4_, and [ReCl(PH_3_)_3_(C_5_H_4_)]-[**Re**]C_5_H_4_.

**Figure 2:**
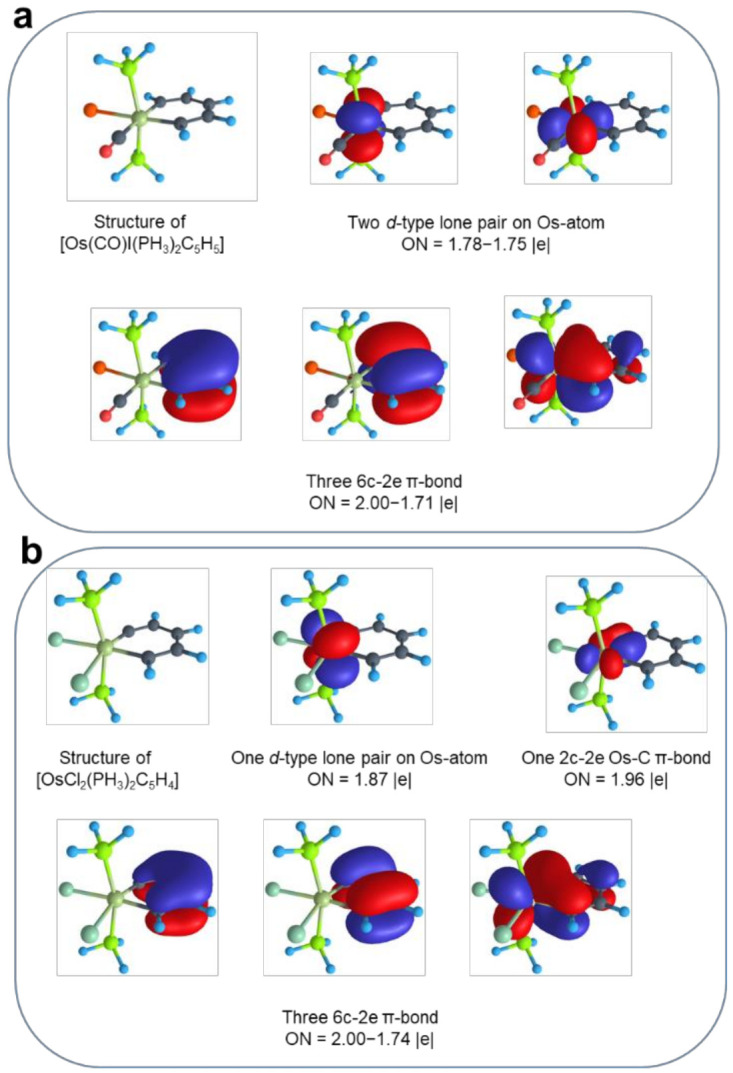
Results of the AdNDP analysis for [Os(CO)I(PH_3_)_2_(C_5_H_5_)] (**a**) and [OsCl_2_(PH_3_)_2_(C_5_H_4_)] (**b**). Only selected bonding elements are shown.

To analyze the chemical bonding in the investigated species, we performed an AdNDP analysis as implemented in AdNDP 2.0 code. We start our discussion with the chemical bonding of the osmabenzene. The [**Os**]C_5_H_5_ complex has 66 valence electrons that could be localized into 33 two-electron bonding elements. Following the idea of AdNDP analysis, the localization starts from the one-center two-electron (1c-2e) elements, or lone pairs. We found two *s*-type lone pairs on oxygen and iodine atoms with occupation numbers (ON) 2.00–1.98 |e|, two *p*-type lone pairs on iodine with ON = 1.99–1.93 |e|, and two *d*-type lone pairs (*d*_yz_ and *d*_x2-y2_) on the Os-atom with ON = 1.78–1.75 |e| (Figure 2a). A consequent localization of 2c-2e bonding elements leads to a σ-bonding framework with sixteen C-O, C-C, C-H, and P-H 2c-2e bonds with ON = 2.00–1.97 |e|. The Osmium atom has a quasi-octahedral environment, and six Os-C, Os-P, and Os-I 2c-2e σ-bonds with ON = 1.98–1.93 |e| can be localized. Predictably, two 2c-2e π-bonds were found in the -C≡O ligand rendering the C-O bond as a triple bond. The remaining six valence electrons form π-delocalized 6c-2e bonds, resembling benzene 6c-2e delocalization (Figure 2a). The shape of those bonds and the number of electrons (in agreement with the Hückel’s [4n+2] rule) render this complex as π-aromatic. The complete bonding pattern of [**Os**]C_5_H_5_ can be found in the Supporting Information file (Figure S1), while the most significant bonding elements are shown in Figure 2a.

We want to note that this picture contradicts with some recent descriptions of metallabenzenes, where eight [3] and ten [38,47] π-electrons were meant to be delocalized in the metallocycle. It was discussed that not only the *d*_xz_ orbital of the Os-atom participates in the formation of π-conjugation, but also the *d*_yz_ orbital overlaps with the π-electrons of the carbon framework. However, this conclusion was made based on the canonical molecular orbital (MO) picture, which is delocalized in its nature. We believe that the picture obtained by the AdNDP is more chemically intuitive and more descriptive since it is based on the matrix representation of the first-order reduced density operator. We can definitely localize the *d*_yz_ lone pair on the Os-atom which does not participate in delocalization in any significant manner, although it is formally of the same symmetry as some orbitals in the π-conjugated system. To show that, we localized this lone pair as a 6c-2e π-bond. The increase in ON was only 0.1 |e| showing a tiny degree of the delocalization of the *d*_yz_ lone pair over the metallabenzene ring. Thus, we believe that the initial description proposed by Thorn and Hofmann is correct, and there is no deviation from Hückel’s aromaticity rule for metallabenzynes/metallabenzenes complexes.

A cognate chemical bonding was found for the [**Os**]C_5_H_4_ complex (Figure S2). The main difference in chemical bonding is the presence of a 2c-2e Os-C in-plane π-bond (Figure 2b), which is also reflected in the significant shortening of Os=C distance with respect to the Os-C single-bond (~0.27 Å difference). A similar chemical bonding pattern was found for rhenabenzyne (Figure S3). It is a very intriguing result, since, to the best of our knowledge, the coexistence of the in-plane π-bond with the π-aromatic system has never been described before in the realms of the AdNDP localization technique. The possibility of such a reconciliation opens up an opportunity to design quite unique structures with doubly aromatic metal-carbon rings that will be described in the next subsection.

### 3.2. Hückel’s [4n+2] σ- and π- Double Aromaticity of Novel Carbon-Metal Structures

**Figure 3:**
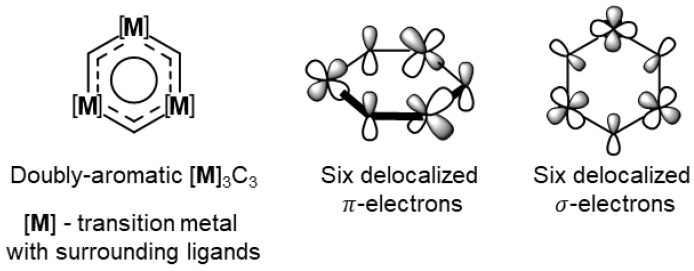
Proposed structure of doubly aromatic metal-carbon ring and orbitals’ representation of conjugated π- and σ- electrons.

The possibility to use the in-plane *p*-orbital of carbon and *d*_x2-y2_-orbital of the transition metal without the introduction of a huge strain (in comparison to the case of the planar benzyne C_6_H_4_ ring, where a highly strained C≡C bond is present) allows proposing possible metal-carbon structures that will employ delocalization not only in the π-fashion but also in the σ-regime. As illustrated in Figure 3, the combination of three carbon atoms and three transition metal atoms could potentially produce a stable doubly aromatic six-membered ring.

**Figure 4:**
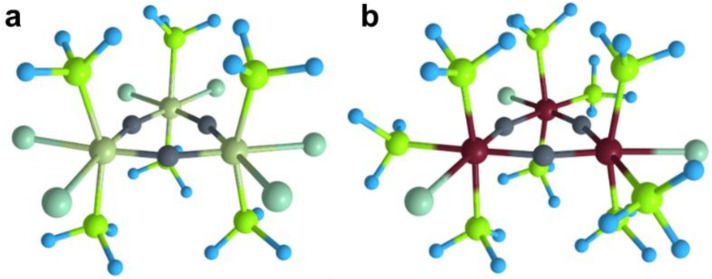
Optimized structures of proposed doubly aromatic complexes: [**Os**]_3_C_3_ (**a**) and [**Re**]_3_C_3_ (**b**).

To test this hypothesis, we employed the [**M**] motifs ([**M**]: [OsCl_2_(PH_3_)_2_], [ReCl(PH_3_)_3_]) that were used in the first part of this work and combined them with three carbon atoms to form a six-membered ring. As a result, [{OsCl_2_(PH_3_)_2_}_3_C_3_] ([**Os**]_3_C_3_) and [{ReCl(PH_3_)_3_}_3_C_3_] ([**Re**]_3_C_3_) stoichiometries were obtained. The optimized structures are found to be a local minimum without possessing any imaginary frequencies. The WF was checked to be stable, and similar triplet species are significantly higher in energy than the singlet ones. The geometries of the proposed species are shown in Figure 4. Remarkably, both structures contain the M_3_C_3_ six-membered planar ring.

**Figure 5:**
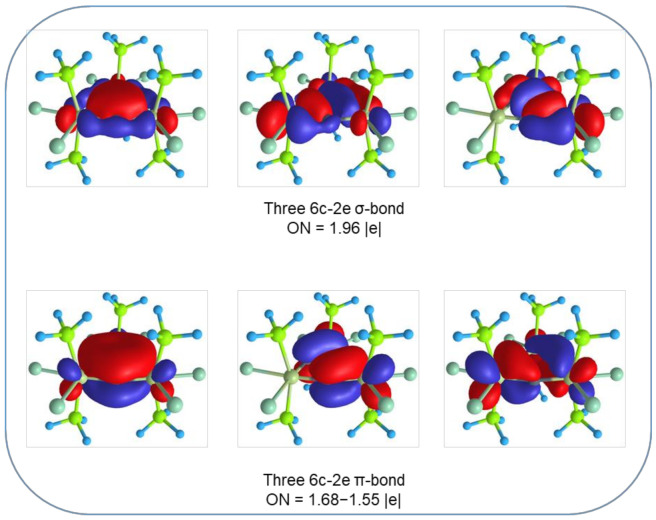
Results of the AdNDP analysis for [**Os**]_3_C_3_. Only σ- and π- delocalized bonding elements are shown. The cognate pattern was found for [**Re**]_3_C_3_.

The AdNDP analysis reveals the cognate chemical bonding pattern as was found in metallabenzynes. The localized lone pairs and σ-bonding framework coincide (Figures S4 and S5). However, the remaining 12 electrons form three delocalized 6c-2e σ- and π-bonding elements (Figure 5). The shape of those elements agrees with the proposed model bonding depicted in Figure 3. We note that the occupation numbers of π-aromatic bonds are rather low (1.68–1.55 |e| for [**Os**]_3_C_3_ and 1.79–1.74 |e| for [**Re**]_3_C_3_); however, the occupancies could be increased up to 2.00–1.98 |e| values by including phosphorous atoms in the localization scheme (resulting in 12c-2e bonds). Thus, 6c-2e π-bonds show some delocalization character over -PH_3_ ligands. Previously, the presence of double aromaticity in metal systems was discussed for M_4_^2−^ molecules composed out of Al, Ga, and In atoms [64,65]. Moreover, recently, Saito and coworkers synthesized a molecule ([C_6_(SePh)_6_][SbF_6_]_2_) in an isolated form that possesses a double aromaticity arising from σ- and π-rings [78].

To assess the aromaticity of metallo-organic rings with a quantitative parameter, we calculated NICS_zz_(0) and NICS_zz_(1) indices (Table 1) at the center of the aromatic rings. The exact coordinates of the special points for each cluster can be found in the Supporting Information file (Table S2). The significantly low values of NICS_zz_(0) and NICS_zz_(1) indices inside of aromatic rings and the increasing of the NICS_zz_(0) values for [**M**]_3_C_3_ species are in agreement with the overall description of double aromaticity in the proposed structures and π-aromaticity of the metallabenzenes and metallabenzynes species.

We note that the difference in the trends for NICS_zz_(0) and NICS_zz_(1) for [**M**]C_5_H_4_ and [M]_3_C_3_ is due to the different types of aromaticity that are present in those systems. Since [**M**]C_5_H_4_ is a π-aromatic system, we observe that NICS_zz_(0) values are less negative than NICS_zz_(1) values (a similar trend can be found in π-aromatic benzene). In turn, [**M**]_3_C_3_ systems are doubly aromatic and possess σ-aromaticity in addition to π-aromaticity. For σ-aromatic systems, it was found before that NICS_zz_(0) is more negative than NICS_zz_(1) [79,80]. Thus, due to the presence of σ-aromaticity, [**M**]_3_C_3_ systems possess largely negative NICS_zz_(0). Such a trend could be vividly visualized using NICS_zz_-Scan plots (Figure S6). Slightly less negative values of NICS_zz_(1) of [**M**]_3_C_3_ in comparison with [**M**]C_5_H_4_ are due to the delocalization of π-electrons to the -PH_3_ ligands, which is also reflected in the ONs of π-delocalized bonds obtained from the AdNDP analysis.

**Figure 6:**
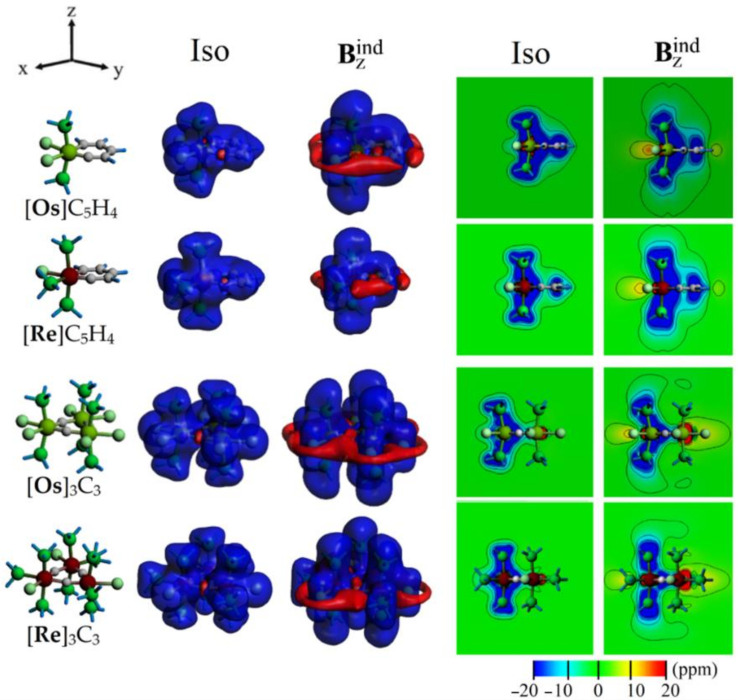
The induced magnetic field for [**Os**]C_5_H_4_, [**Re**]C_5_H_4_, [**Os**]_3_C_3_, and [**Re**]_3_C_3_, given as isosurface (±5 ppm, Blue: Shielding; Red: Deshielding) and contour plot (Side-view; Negative values: Shielding; positive: Deshielding).

To access the spatial distribution of the magnetic shielding parameters, the isosurface representation of the induced magnetic field (B_ind_) was calculated. It accounts for the characteristic patterns of the magnetic response at the molecular surroundings of the complexes, revealing the inherent characteristics given by the above discussed bonding pattern related to the Hückel’s [4n + 2] aromaticity on the studied metallobenzynes and metallabenzenes complexes. The isotropic term is related to the orientational averaged response owing to the molecular tumbling in solution, which is related to NICS given as an isosurface along the overall structure denoted as Iso (Figure 6), which shows a larger contribution from the M-C, M-Cl, and M-P bond (M=Os, Re). In addition, for [**Os**]C_5_H_4_ and [**Re**]C_5_H_4_, a shielding surface at the six membered MC_5_H_4_ ring indicates its aromatic character. In agreement to NICS_zz_(0) and NICS_zz_(1) values (Table 1), the related B_z_^ind^ isosurface exhibits an enhanced shielding response with a complementary deshielding region along the ring backbone, denoting the shielding cone property from aromatic rings. The contour plot for B_z_^ind^ supports this behavior, showing a shielding value of ~ −10 ppm nearby the center of the ring increasing along the *z*-axis, which is enhanced towards the C-C and C-M bonds (>−20 ppm).

**Figure 7:**
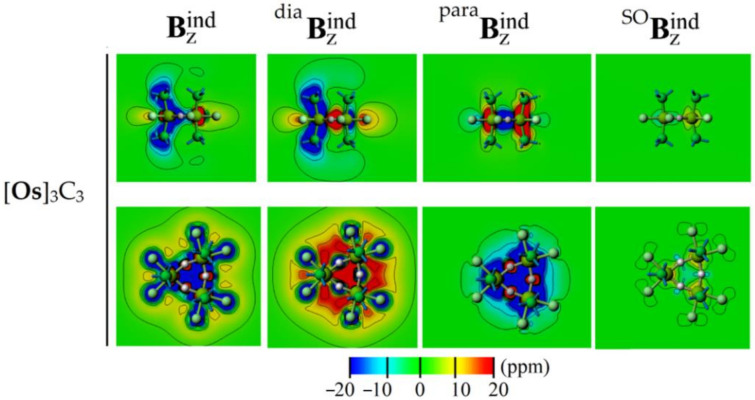
The induced magnetic field for [**Os**]_3_C_3_, given as contour plot and its contribution from diamagnetic (^dia^B_z_^ind^), paramagnetic (^para^B_z_^ind^), and spin–orbit (^SO^B_z_^ind^) terms.

For [**Os**]_3_C_3_ and [**Re**]_3_C_3_ rings, the NICS_zz_(0) values (Table 1) show an enhanced shielding denoting their double aromatic characteristics. Similarly, the isosurface and contour plot representation of Iso and B_z_^ind^ denotes an enhanced shielding response at the center of the M_3_C_3_ ring, in comparison to the MC_5_H_4_ case, which is decreased along the *z*-axis as previously depicted by NICS_zz_(1). Moreover, nearby the M center, a deshielding contribution is found, suggesting that besides the double aromatic character of the M_3_C_3_ ring, the NICS_zz_ values are also affected by the contribution from the metal center, leading to a decrease in the shielding behavior nearby the center of the [**Os**]_3_C_3_ and [**Re**]_3_C_3_ rings, as shown from the side-view contour plot of B_z_^ind^ from Figure 6, but clarified by the top-view from Figure 7. It is important to note that such an observation is exclusive for metal-containing rings in contrast to hydrocarbons or other light element structures because of the contribution from core electrons [81].

Furthermore, the contribution from the metal center to the NICS_zz_ and its related isosurface (B_z_^ind^) terms is explored in terms of diamagnetic (^dia^B_z_^ind^), paramagnetic (^para^B_z_^ind^), and spin–orbit (^SO^B_z_^ind^) contributions within the two components’ ZORA/GIAO formalism [82].

B_z_^ind^ = ^dia^B_z_^ind^ + ^para^B_z_^ind^ + ^SO^B_z_^ind^

The diamagnetic contribution involves an unperturbed electron density and is largely affected by the number of core electrons, thus increasing according to the atomic number (*Z*) of the involved metal centers [82]. The paramagnetic term (^para^B_z_^ind^) involves the mixing of ground and excited states upon the external magnetic field, and is mainly affected by the frontier orbital structure, and thus accounting for the classical electron counting consideration, such as Hückel’s rule. Lastly, the spatial contribution from the spin-orbit coupling is accounted by the ^SO^B_z_^ind^ term [83].

From Figure 7, the strong shielding behavior at the center of the Os_3_C_3_ ring is contributed from the paramagnetic term, i.e., accounting for the favorable double aromatic character, as depicted by the bonding pattern fulfilling both in-plane and out-of-plane Hückel circuits. Interestingly, the diamagnetic contribution shows a large deshielding contribution originated at each M center, with a small ^SO^B_z_^ind^ contribution. Hence, the contribution from core electrons from M decreases the long range characteristics of the shielding cone property [84] of the Os_3_C_3_ ring. Similar results were obtained for [**Re**]_3_C_3_ (Figure S7).

## 4. Conclusions

In this research we showed that metallobenzynes and metallabenzenes complexes possess Hückel’s [4n + 2] classical π-aromatic conjugated system. We showed that the previous assignment of eight and ten delocalized π-electrons does not agree with the AdNDP localization picture, and the *d*_yz_ lone pair of a transition metal has an appreciable occupation number and cannot be considered as a delocalized 6c-2e bond. By analyzing chemical bonding of metallabenzynes, we firstly described an in-plane π-bond coexisting with π-aromaticity in realms on the AdNDP analysis. It allowed us to construct two novel molecules ([**Os**]_3_C_3_ and [**Re**]_3_C_3_) with doubly aromatic six-membered ring, denoting the contribution from the metal core electrons to the magnetic characteristics of the overall structure. We believe these findings will help in the development and understanding of chemical bonding in metallocycles and open a possibility to discover a novel class of doubly aromatic carbon-metal species.

## Figures and Tables

**Table 1:** NICS_zz_(0) and NICS_zz_(1) indices calculated for complexes investigated in this work.

Complex	NICS_zz_(0)	NICS_zz_(1)
[**Os**]C_5_H_4_	−9.2	−15.0
[**Re**]C_5_H_4_	−9.9	−14.2
[**Os**]_3_C_3_	−23.5	−2.8
[**Re**]_3_C_3_	−29.5	−6.3

## Data Availability

The data that support the findings of this study are available from the corresponding authors on a reasonable request.

## Supplementary Materials

Figure S1: Chemical bonding pattern of [**Os**]C_5_H_5_, Figure S2: Chemical bonding pattern of [**Os**]C_5_H_4_, Figure S3: Chemical bonding pattern of [**Re**]C_5_H_4,_ Figure S4: Chemical bonding pattern of [**Os**]_3_C_3,_ Figure S5: Chemical bonding pattern of [**Re**]_3_C_3,_ Figure S6: The NICS_zz_ values calculated at different distances from the ring center, Figure S7: The induced magnetic field for investigated complexes, Table S1: Optimized structures of investigated complexes, Table S2: Coordinates of chosen points for NICS calculation.
