# Supplementary material for: Occurrence of Double Bond in π-Aromatic Rings: An Easy Way to Design Doubly Aromatic Carbon-Metal Structures
Source: Molecules. 2021 Nov 29;26(23):7232. doi: 10.3390/molecules26237232 (PMC8659221; doi:10.3390/molecules26237232)
Supplement: Supplementary file 1 [file molecules-26-07232-s001.zip › molecules-1476161-supplementary.pdf]

Occurrence of Double Bond in π−Aromatic Rings: An Easy Way to Design Doubly Aromatic Carbon−Metal Structures

Nikolay V. Tkachenko [1], Alvaro Muñoz-Castro [2] and Alexander I. Boldyrev [1,*]

[1] Department of Chemistry and Biochemistry, Utah State University, 0300 Old Main Hill, Logan, UT 84322-0300, USA; nikolay.tkachenko95@gmail.com
[2] Grupo de Química Inorgánica y Materiales Moleculares, Facultad de Ingeniería, Universidad Autonoma de Chile, El Llano Subercaseaux, Santiago 2801, Chile; alvaro.munoz@uautonoma.cl
* Correspondence: a.i.boldyrev@usu.edu

Supporting Information

Contents

1. Figure S1. Chemical bonding pattern of [**Os**]C_5H_5 .... S2
2. Figure S2. Chemical bonding pattern of [**Os**]C_5H_4 .... S3
3. Figure S3. Chemical bonding pattern of [**Re**]C_5H_4 .... S4
4. Figure S4. Chemical bonding pattern of [**Os**]$_3C_3$ S5
5. Figure S5. Chemical bonding pattern of [**Re**]$_3C_3$ S5
6. Figure S6. The $NICS_{zz}$-Scan plots S6
7. Figure S7. The induced magnetic field for investigated complexes....................... S7
8. Table S1. Optimized structures of investigated complexes.................................. S8
9. Table S2. Coordinates of chosen points for NICS calculation. S11

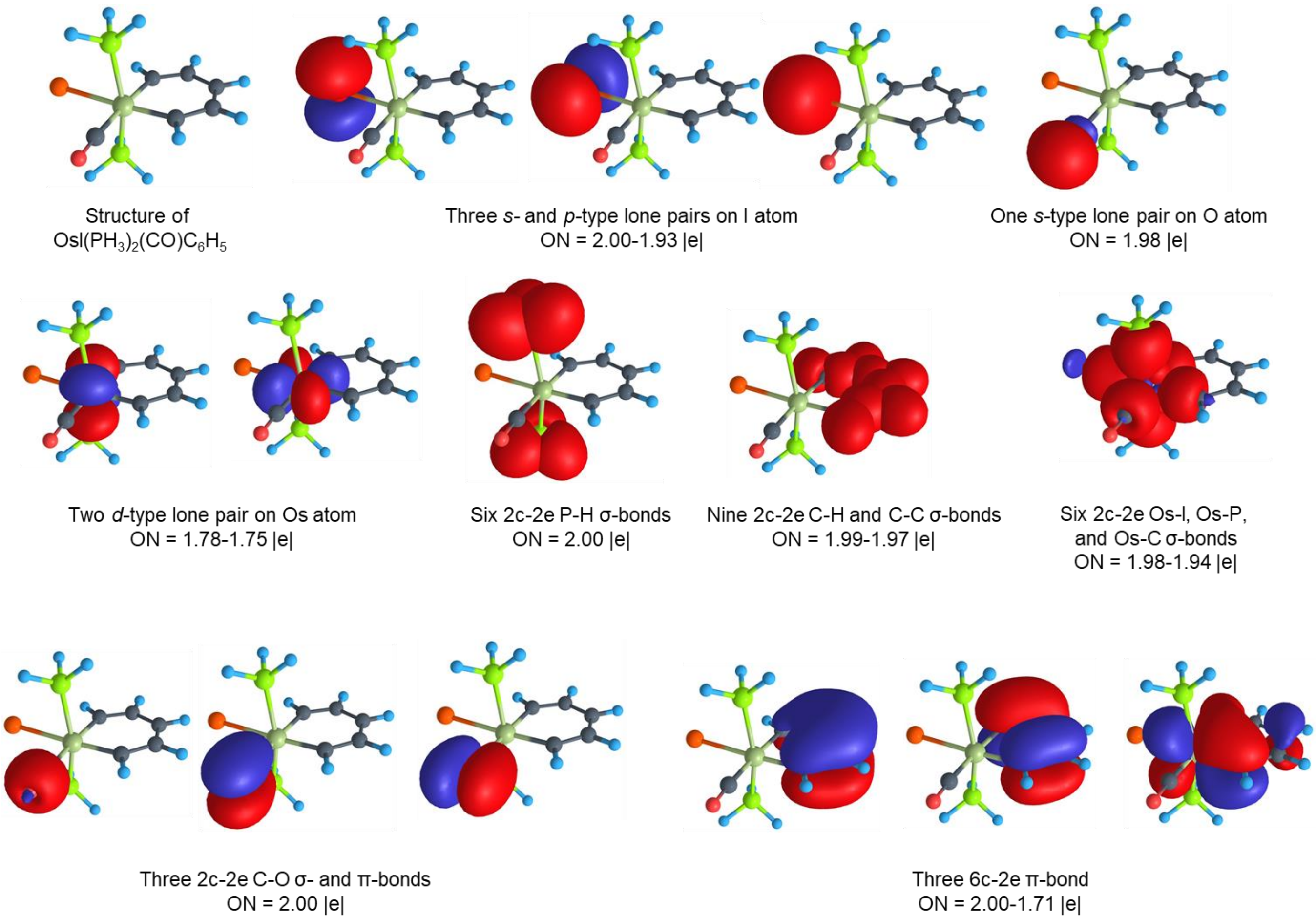

Figure S1. Chemical bonding pattern of [**Os**]C_5H_5.

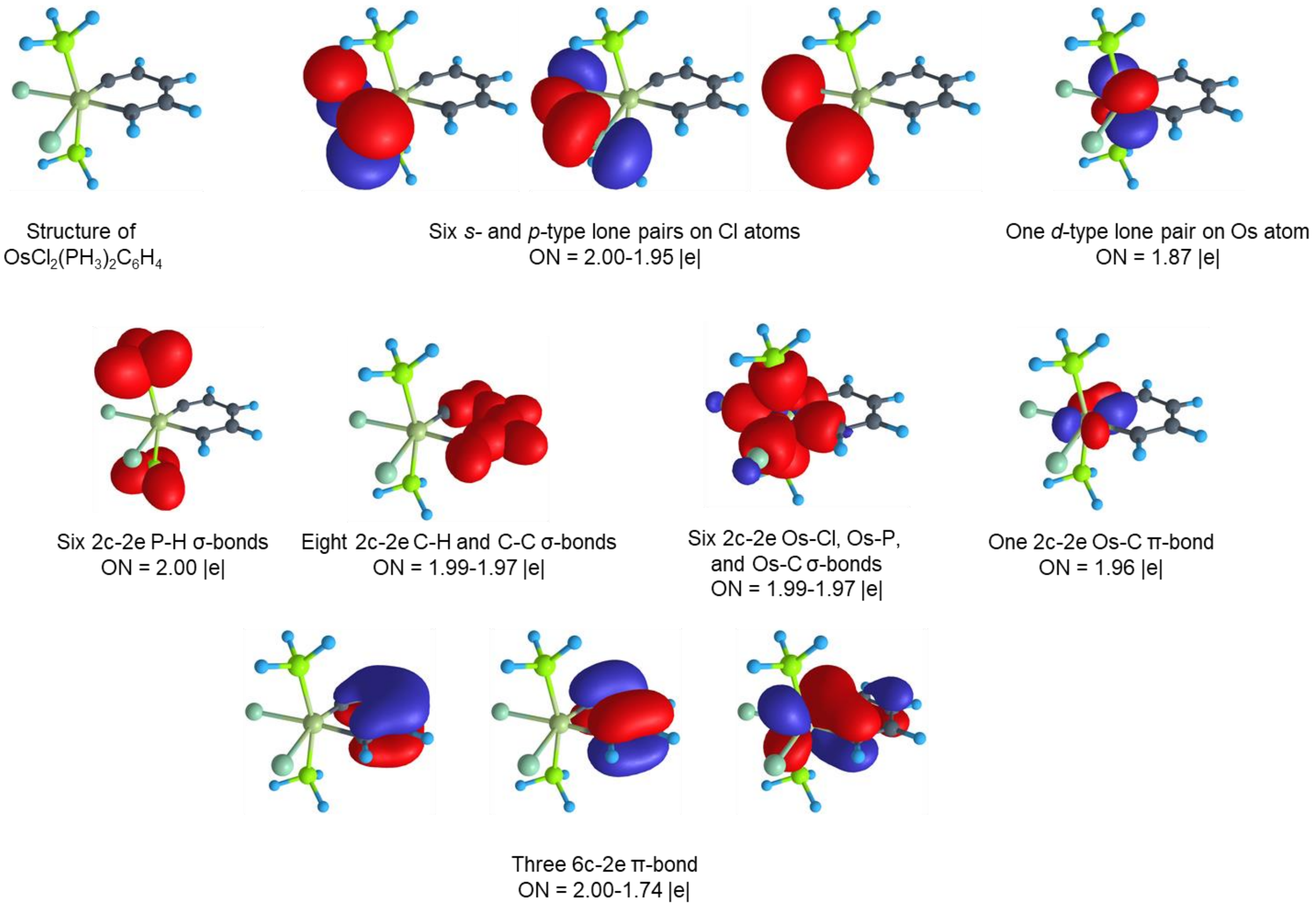

Figure S2. Chemical bonding pattern of [**Os**]C_5H_4.

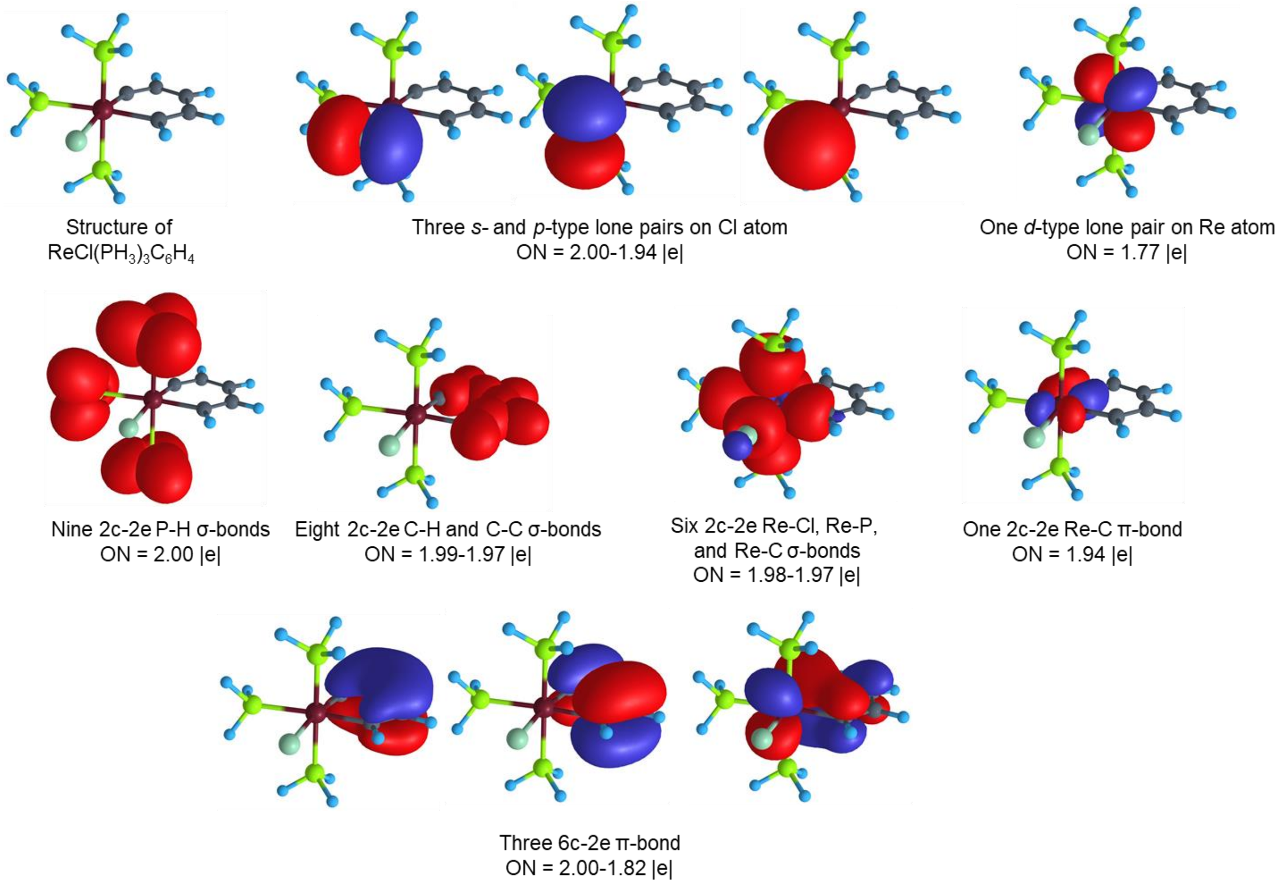

Figure S3. Chemical bonding pattern of [**Re**]C_5H_4.

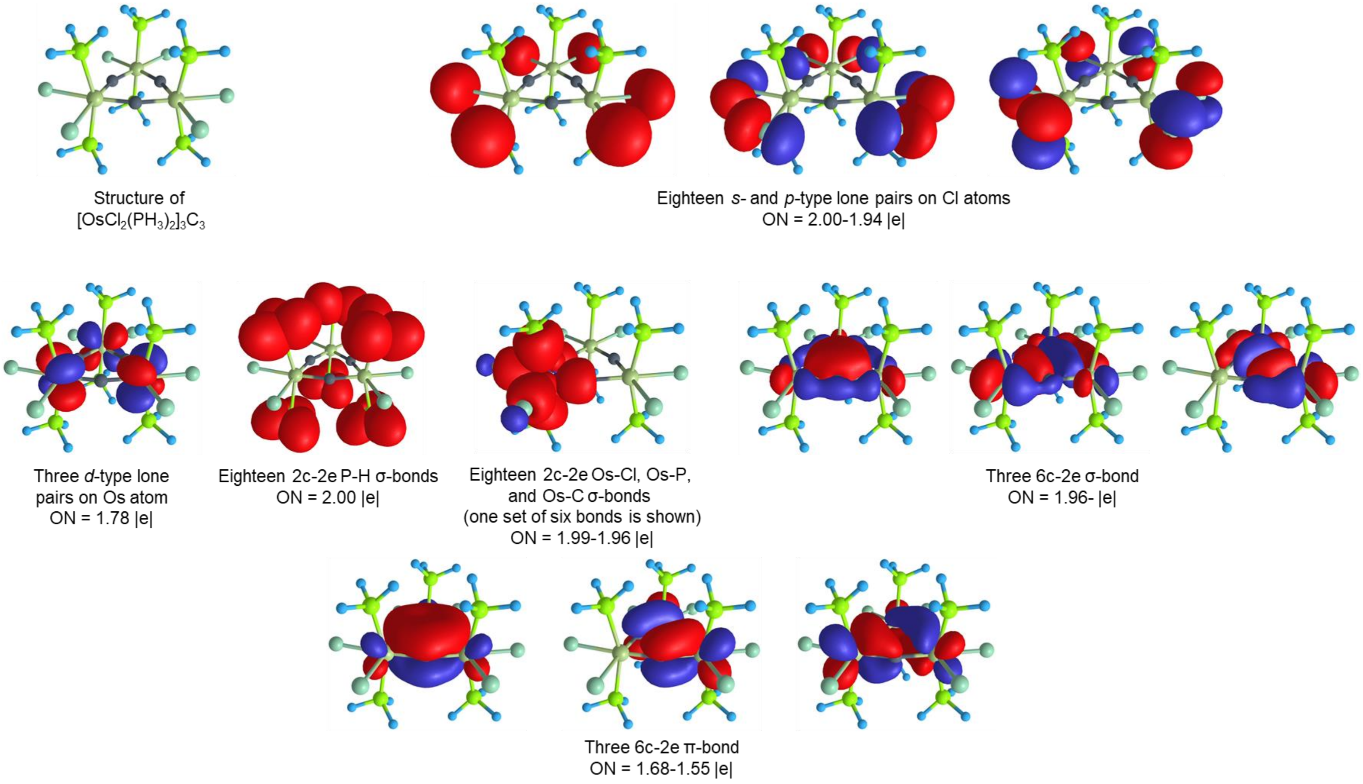

Figure S4. Chemical bonding pattern of [**Os**]$_3$C$_3$.

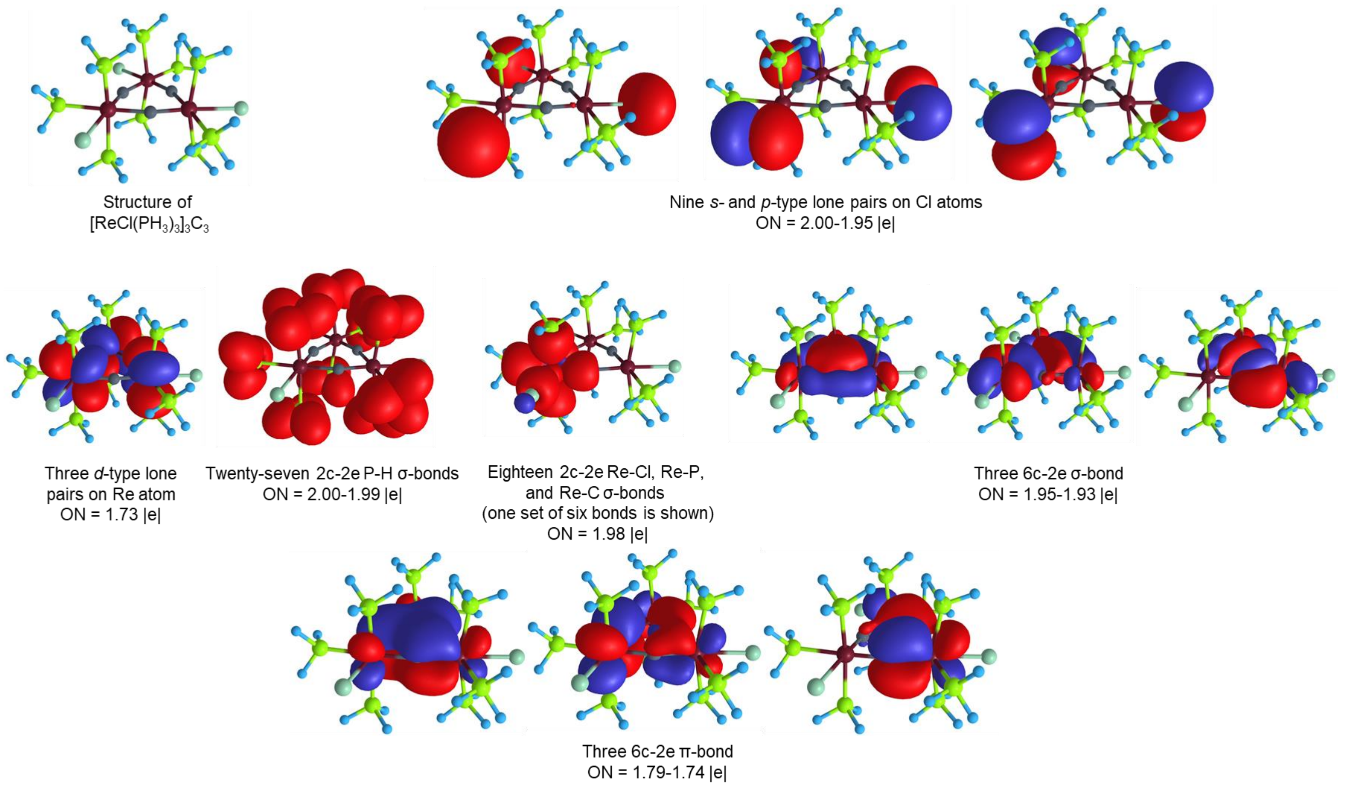

Figure S5. Chemical bonding pattern of [**Re**]$_3$C$_3$.

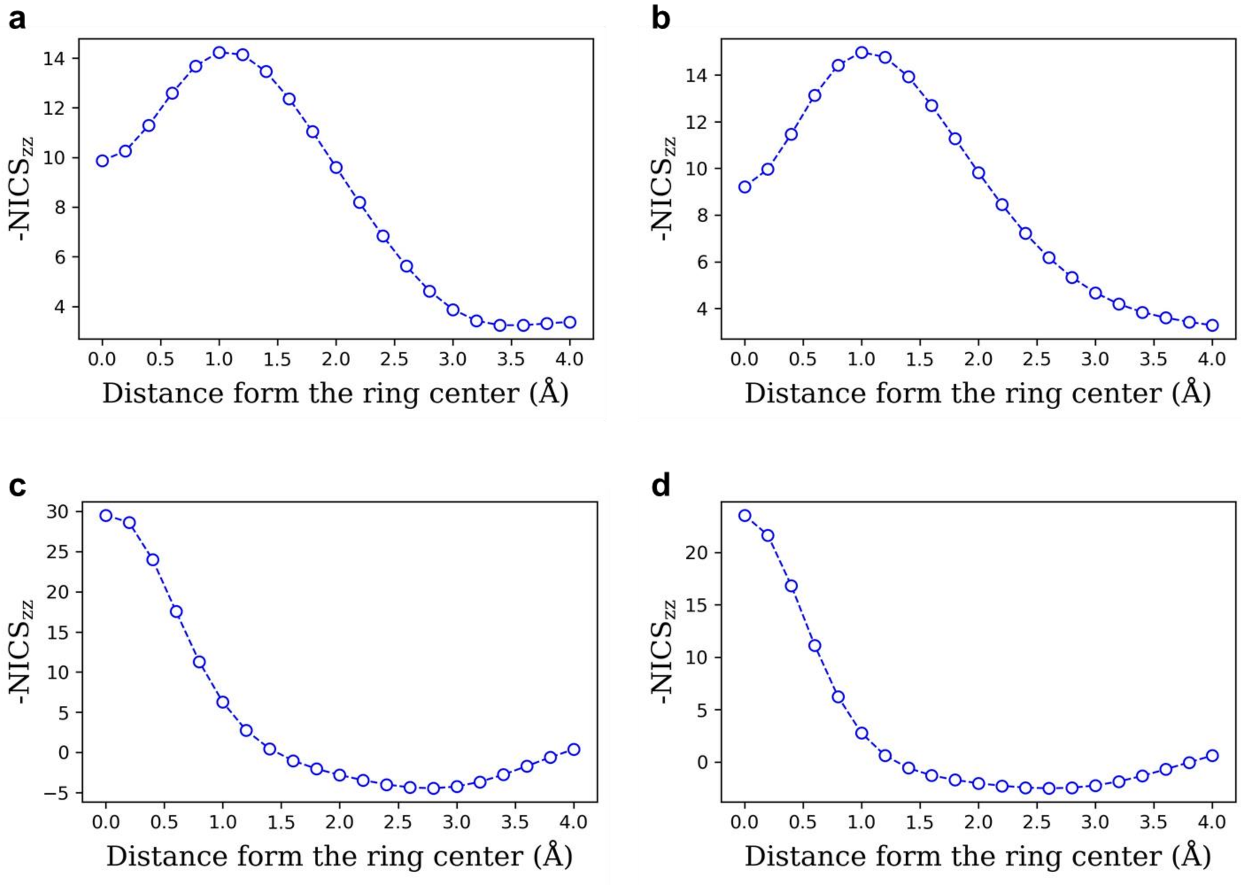

Figure S6. The NICS$_{zz}$ values calculated at different distances from the ring center: [**Re**]C_5H_4 (**a**), [**Os**]C_5H_4 (**b**), [**Re**]C_3 (**c**), [**Os**]C_3 (**d**).

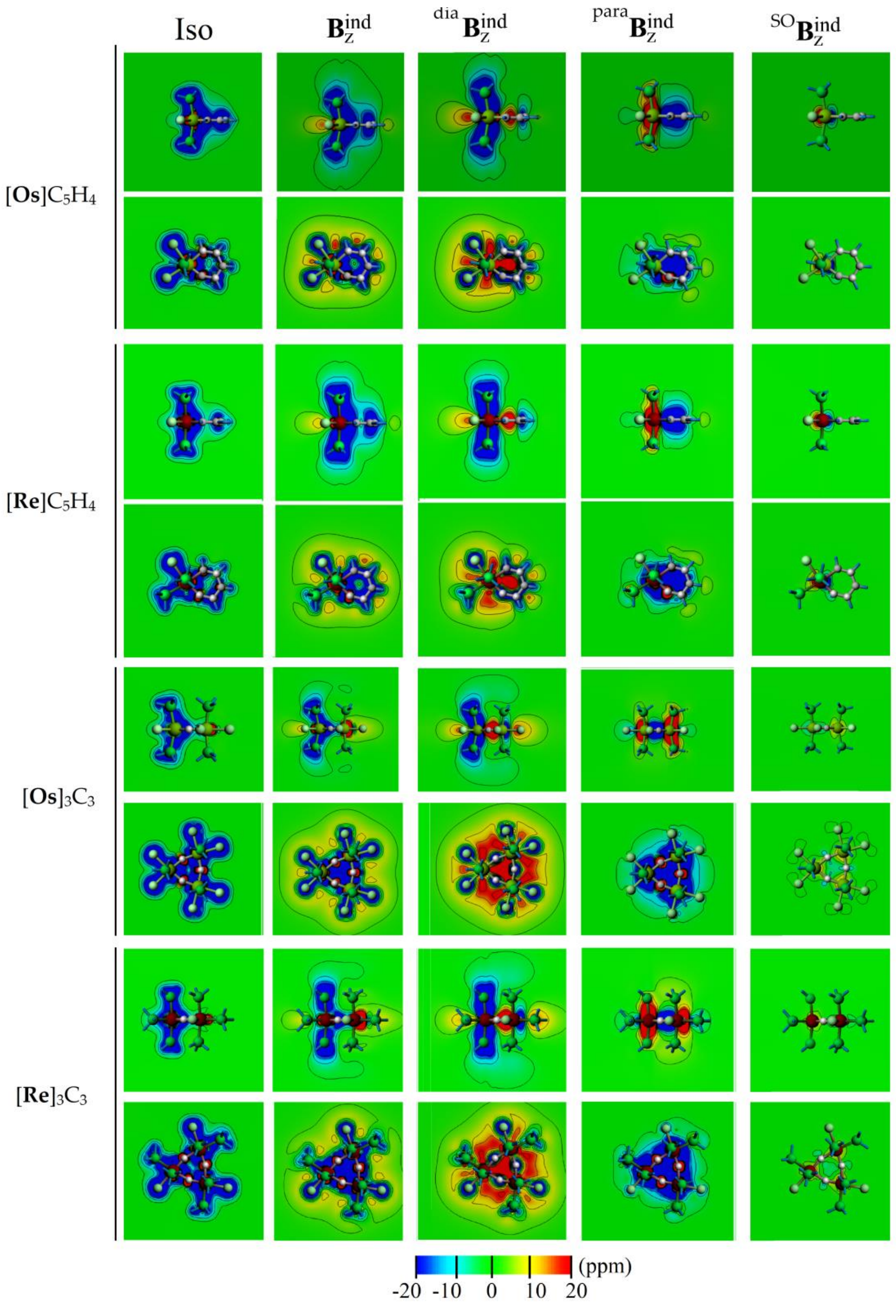

Figure S7. The induced magnetic field for investigated complexes, given as contour plot and its contribution from diamagnetic ($^{dia}B_z^{ind}$), paramagnetic ($^{para}B_z^{ind}$), and spin–orbit ($^{SO}B_z^{ind}$) terms.

Table S1. Optimized structures of investigated complexes.

[Os]C_5H_5	PBE0/def2tzvp, 0 Imaginary Frequencies
	76 −0.016377000 0.510429000 0.000000000<br>6 1.978674000 −0.093854000 0.000000000<br>6 0.653674000 2.366970000 0.000000000<br>1 −0.074399000 3.184241000 0.000000000<br>6 3.116042000 0.664446000 0.000000000<br>6 1.963773000 2.856527000 0.000000000<br>1 4.093176000 0.185347000 0.000000000<br>1 2.107521000 3.934684000 0.000000000<br>6 3.098304000 2.072589000 0.000000000<br>1 4.058259000 2.584156000 0.000000000<br>53 −0.786554000 −2.224369000 0.000000000<br>15 −0.016377000 0.098443000 2.293886000<br>15 −0.016377000 0.098443000 −2.293886000<br>1 0.833143000 −0.931161000 −2.733990000<br>1 −1.224690000 −0.297518000 −2.895292000<br>1 0.370275000 1.117437000 −3.194957000<br>1 0.833143000 −0.931161000 2.733990000<br>1 −1.224690000 −0.297518000 2.895292000<br>1 0.370275000 1.117437000 3.194957000<br>1 2.168296000 −1.172740000 0.000000000<br>6 −1.815920000 1.187062000 0.000000000<br>8 −2.856776000 1.666256000 0.000000000
[**Os**]C_5H_4	PBE0/def2tzvp, 0 imaginary frequencies
	76 0.057262000 −0.018753000 −0.087455000<br>6 −1.345279000 −0.272567000 −1.124134000<br>6 −1.429875000 0.250012000 1.266407000<br>1 −1.094521000 0.475854000 2.278966000<br>6 −2.700536000 −0.330860000 −1.309748000<br>6 −2.795036000 0.186883000 1.092826000<br>1 −3.175186000 −0.540349000 −2.259646000<br>1 −3.447335000 0.358006000 1.944353000<br>6 −3.410720000 −0.090924000 −0.141615000<br>1 −4.496263000 −0.118858000 −0.186055000<br>17 2.097236000 −0.354440000 −1.395549000<br>17 1.507082000 0.517560000 1.835950000<br>15 0.381145000 2.278237000 −0.438918000<br>15 0.497784000 −2.279395000 0.360778000<br>1 0.607676000 −3.134701000 −0.749295000<br>1 1.729813000 −2.479110000 1.001468000<br>1 −0.361658000 −3.038300000 1.183799000<br>1 −0.263203000 2.929735000 −1.511558000<br>1 1.721405000 2.611664000 −0.688507000<br>1 0.058692000 3.150372000 0.615917000

[**Re**]C_5H_4	PBE0/def2tzvp, 0 imaginary frequencies
	75 −0.083604000 −0.000001000 −0.121317000<br>6 1.311462000 −0.000040000 −1.225144000<br>6 1.474551000 −0.000068000 1.281708000<br>1 1.194326000 −0.000004000 2.339765000<br>6 2.687335000 −0.000068000 −1.423624000<br>6 2.824570000 −0.000064000 1.044917000<br>1 3.163564000 −0.000076000 −2.397324000<br>1 3.517918000 −0.000056000 1.883419000<br>6 3.410784000 −0.000054000 −0.253136000<br>1 4.495927000 −0.000058000 −0.320386000<br>17 −1.708853000 0.000042000 1.811478000<br>15 −0.209051000 −2.348941000 0.219419000<br>15 −0.208857000 2.348945000 0.219431000<br>1 −1.398180000 3.063042000 −0.057110000<br>1 −0.009341000 2.795688000 1.541614000<br>1 0.698718000 3.204069000 −0.441353000<br>1 0.698394000 −3.204154000 −0.441430000<br>1 −1.398466000 −3.062909000 −0.057053000<br>1 −0.009495000 −2.795717000 1.541585000<br>1 −3.044141000 1.053907000 −1.412807000<br>1 −1.970206000 0.000043000 −2.916906000<br>1 −3.044241000 −1.053680000 −1.412778000<br>15 −2.103837000 0.000067000 −1.511778000
[**Os**]$_3C_3$	PBE0/def2tzvp, 0 imaginary frequencies
	76 0.000000000 2.103220000 −0.000108000<br>6 1.183242000 0.683137000 −0.000277000<br>6 −1.183235000 0.683149000 −0.000277000<br>6 −0.000007000 −1.366286000 −0.000277000<br>17 1.710170000 3.877612000 0.096341000<br>17 −1.710185000 3.877631000 −0.095748000<br>15 −0.148940000 2.563993000 −2.306385000<br>15 0.149220000 2.562665000 2.306355000<br>1 1.428475000 2.503150000 2.883775000<br>1 −0.255791000 3.870791000 2.607823000<br>1 −0.593270000 1.832276000 3.262586000<br>1 0.594889000 1.835124000 −3.262706000<br>1 0.254883000 3.872752000 −2.606656000<br>1 −1.427798000 2.503637000 −2.884612000<br>76 1.821442000 −1.051610000 −0.000108000<br>1 1.291820000 −1.432751000 −3.262706000<br>1 3.226460000 −2.157111000 −2.606656000<br>1 2.882112000 −0.015309000 −2.884612000<br>1 1.453554000 −2.488671000 2.883775000<br>1 3.480099000 −1.713874000 2.607823000

	1 1.883433000 −0.402351000 3.262586000<br>15 2.144723000 −1.410561000 2.306355000<br>17 4.213219000 −0.457752000 −0.095748000<br>17 2.503025000 −3.419856000 0.096341000<br>15 2.294953000 −1.153010000 −2.306385000<br>76 −1.821442000 −1.051610000 −0.000108000<br>1 −1.886709000 −0.402373000 −3.262706000<br>1 −3.481343000 −1.715641000 −2.606656000<br>1 −1.454314000 −2.488328000 −2.884612000<br>1 −2.882029000 −0.014479000 2.883775000<br>1 −3.224307000 −2.156917000 2.607823000<br>1 −1.290162000 −1.429925000 3.262586000<br>15 −2.293943000 −1.152104000 2.306355000<br>17 −2.503034000 −3.419879000 −0.095748000<br>17 −4.213195000 −0.457755000 0.096341000<br>15 −2.146013000 −1.410983000 −2.306385000
[**Re**]$_3$C$_3$	PBE0/def2tzvp, 0 imaginary frequencies
	75 0.488616000 −2.112701000 0.027325000<br>6 −0.970561000 −1.014492000 0.046660000<br>6 1.363927000 −0.333281000 0.046650000<br>6 −0.393283000 1.347878000 0.046299000<br>17 2.512902000 −3.678693000 −0.161736000<br>15 0.859504000 −2.263191000 −2.323218000<br>15 0.785945000 −2.275262000 2.371450000<br>1 −0.177257000 −2.840369000 3.243127000<br>1 1.892743000 −3.055001000 2.773192000<br>1 1.056312000 −1.123606000 3.146294000<br>1 0.084050000 −1.555896000 −3.273557000<br>1 0.796501000 −3.531743000 −2.940866000<br>1 2.134856000 −1.880390000 −2.784573000<br>1 −0.591603000 −5.205598000 1.004299000<br>1 −2.169290000 −4.232527000 −0.032315000<br>1 −0.565268000 −5.140735000 −1.101074000<br>15 −0.757515000 −4.245088000 −0.021914000<br>75 1.585427000 1.479532000 0.027194000<br>17 1.929900000 4.015593000 −0.162669000<br>15 1.531263000 1.875468000 −2.323473000<br>15 1.576136000 1.818733000 2.371193000<br>15 4.055261000 1.466443000 −0.020538000<br>1 1.307782000 0.849853000 −3.273730000<br>1 2.661280000 2.455976000 −2.940459000<br>1 0.561537000 2.787591000 −2.785622000

	1 2.546867000 1.267869000 3.243642000<br>1 1.696966000 3.167224000 2.772870000<br>1 0.443247000 1.476449000 3.145252000<br>1 4.803575000 2.092104000 1.005017000<br>1 4.750136000 0.237450000 −0.028387000<br>1 4.735552000 2.078756000 −1.100365000<br>75 −2.073936000 0.633253000 0.027166000<br>17 −4.442560000 −0.336476000<br>−0.161612000<br>15 −2.390017000 0.386736000 −2.323310000<br>15 −2.363042000 0.457194000 2.371266000<br>15 −3.298160000 2.778285000 −0.022026000<br>1 −1.389801000 0.704475000 −3.273791000<br>1 −3.457233000 1.075253000 −2.941088000<br>1 −2.696057000 −0.909320000 −2.784241000<br>1 −2.371463000 1.574023000 3.242804000<br>1 −3.591336000 −0.112102000 2.773248000<br>1 −1.500330000 −0.352189000 3.146152000<br>1 −2.581884000 3.994936000 −0.032592000<br>1 −4.170356000 3.059218000 −1.100937000<br>1 −4.212763000 3.114396000 1.004492000

Table S2. Coordinates of chosen points for NICS calculation.

[**Os**]C_5H_4	76 0.054040000 −0.099087000 −0.119586000<br>6 −1.363000000 −0.049669000 −1.166392000<br>6 −1.413437000 −0.076443000 1.281397000<br>1 −1.063813000 −0.099986000 2.313846000<br>6 −2.720237000 −0.004018000 −1.340739000<br>6 −2.780744000 −0.037769000 1.117828000<br>1 −3.207986000 0.027529000 −2.306446000<br>1 −3.420892000 −0.034143000 1.995366000<br>6 −3.413713000 −0.002240000 −0.138427000<br>1 −4.499544000 0.029074000 −0.172306000<br>17 2.070798000 −0.221871000 −1.499042000<br>17 1.535089000 −0.075665000 1.854000000<br>15 0.483578000 2.201174000 0.050372000<br>15 0.390153000 −2.419934000 −0.198145000<br>1 0.455453000 −3.006885000 −1.473728000<br>1 1.614063000 −2.813811000 0.363255000<br>1 −0.500521000 −3.305744000 0.445270000<br>1 −0.133909000 3.105904000 −0.838651000<br>1 1.836950000 2.522106000 −0.137119000<br>1 0.206517000 2.826453000 1.279004000<br>Point 1 −1.937000000 0.000000000 −0.051000000<br>Point 2 −1.937000000 1.000000000 −0.051000000

[**Re**]C_5H_4	75 −0.083604000 −0.000001000 −0.121317000<br>6 1.311462000 −0.000040000 −1.225144000<br>6 1.474551000 −0.000068000 1.281708000<br>1 1.194326000 −0.000004000 2.339765000<br>6 2.687335000 −0.000068000 −1.423624000<br>6 2.824570000 −0.000064000 1.044917000<br>1 3.163564000 −0.000076000 −2.397324000<br>1 3.517918000 −0.000056000 1.883419000<br>6 3.410784000 −0.000054000 −0.253136000<br>1 4.495927000 −0.000058000 −0.320386000<br>17 −1.708853000 0.000042000 1.811478000<br>15 −0.209051000 −2.348941000 0.219419000<br>15 −0.208857000 2.348945000 0.219431000<br>1 −1.398180000 3.063042000 −0.057110000<br>1 −0.009341000 2.795688000 1.541614000<br>1 0.698718000 3.204069000 −0.441353000<br>1 0.698394000 −3.204154000 −0.441430000<br>1 −1.398466000 −3.062909000 −0.057053000<br>1 −0.009495000 −2.795717000 1.541585000<br>1 −3.044141000 1.053907000 −1.412807000<br>1 −1.970206000 0.000043000 −2.916906000<br>1 −3.044241000 −1.053680000 −1.412778000<br>15 −2.103837000 0.000067000 −1.511778000<br>Point 1 1.937516000 −0.000049000 −0.116099000<br>Point 2 1.938000000 1.000000000 −0.116000000
[**Os**]$_3C_3$	76 0.000000000 2.103220000 −0.000108000<br>6 1.183242000 0.683137000 −0.000277000<br>6 −1.183235000 0.683149000 −0.000277000<br>6 −0.000007000 −1.366286000 −0.000277000<br>17 1.710170000 3.877612000 0.096341000<br>17 −1.710185000 3.877631000 −0.095748000<br>15 −0.148940000 2.563993000 −2.306385000<br>15 0.149220000 2.562665000 2.306355000<br>1 1.428475000 2.503150000 2.883775000<br>1 −0.255791000 3.870791000 2.607823000<br>1 −0.593270000 1.832276000 3.262586000<br>1 0.594889000 1.835124000 −3.262706000<br>1 0.254883000 3.872752000 −2.606656000<br>1 −1.427798000 2.503637000 −2.884612000<br>76 1.821442000 −1.051610000 −0.000108000<br>1 1.291820000 −1.432751000 −3.262706000<br>1 3.226460000 −2.157111000 −2.606656000<br>1 2.882112000 −0.015309000 −2.884612000<br>1 1.453554000 −2.488671000 2.883775000<br>1 3.480099000 −1.713874000 2.607823000<br>1 1.883433000 −0.402351000 3.262586000

	15 2.144723000 −1.410561000 2.306355000<br>17 4.213219000 −0.457752000 −0.095748000<br>17 2.503025000 −3.419856000 0.096341000<br>15 2.294953000 −1.153010000 −2.306385000<br>76 −1.821442000 −1.051610000 −0.000108000<br>1 −1.886709000 −0.402373000 −3.262706000<br>1 −3.481343000 −1.715641000 −2.606656000<br>1 −1.454314000 −2.488328000 −2.884612000<br>1 −2.882029000 −0.014479000 2.883775000<br>1 −3.224307000 −2.156917000 2.607823000<br>1 −1.290162000 −1.429925000 3.262586000<br>15 −2.293943000 −1.152104000 2.306355000<br>17 −2.503034000 −3.419879000 −0.095748000<br>17 −4.213195000 −0.457755000 0.096341000<br>15 −2.146013000 −1.410983000 −2.306385000<br>Point 1 0.000000000 0.000000000 −0.000193000<br>Point 2 0.000000000 0.000000000 1.000000000
[**Re**]$_3$C$_3$	75 0.488616000 −2.112701000 0.027325000<br>6 −0.970561000 −1.014492000 0.046660000<br>6 1.363927000 −0.333281000 0.046650000<br>6 −0.393283000 1.347878000 0.046299000<br>17 2.512902000 −3.678693000 −0.161736000<br>15 0.859504000 −2.263191000 −2.323218000<br>15 0.785945000 −2.275262000 2.371450000<br>1 −0.177257000 −2.840369000 3.243127000<br>1 1.892743000 −3.055001000 2.773192000<br>1 1.056312000 −1.123606000 3.146294000<br>1 0.084050000 −1.555896000 −3.273557000<br>1 0.796501000 −3.531743000 −2.940866000<br>1 2.134856000 −1.880390000 −2.784573000<br>1 −0.591603000 −5.205598000 1.004299000<br>1 −2.169290000 −4.232527000 −0.032315000<br>1 −0.565268000 −5.140735000 −1.101074000<br>15 −0.757515000 −4.245088000 −0.021914000<br>75 1.585427000 1.479532000 0.027194000<br>17 1.929900000 4.015593000 −0.162669000<br>15 1.531263000 1.875468000 −2.323473000<br>15 1.576136000 1.818733000 2.371193000<br>15 4.055261000 1.466443000 −0.020538000<br>1 1.307782000 0.849853000 −3.273730000<br>1 2.661280000 2.455976000 −2.940459000<br>1 0.561537000 2.787591000 −2.785622000<br>1 2.546867000 1.267869000 3.243642000<br>1 1.696966000 3.167224000 2.772870000<br>1 0.443247000 1.476449000 3.145252000<br>1 4.803575000 2.092104000 1.005017000

	1 4.750136000 0.237450000 −0.028387000<br>1 4.735552000 2.078756000 −1.100365000<br>75 −2.073936000 0.633253000 0.027166000<br>17 −4.442560000 −0.336476000 −0.161612000<br>15 −2.390017000 0.386736000 −2.323310000<br>15 −2.363042000 0.457194000 2.371266000<br>15 −3.298160000 2.778285000 −0.022026000<br>1 −1.389801000 0.704475000 −3.273791000<br>1 −3.457233000 1.075253000 −2.941088000<br>1 −2.696057000 −0.909320000 −2.784241000<br>1 −2.371463000 1.574023000 3.242804000<br>1 −3.591336000 −0.112102000 2.773248000<br>1 −1.500330000 −0.352189000 3.146152000<br>1 −2.581884000 3.994936000 −0.032592000<br>1 −4.170356000 3.059218000 −1.100937000<br>1 −4.212763000 3.114396000 1.004492000<br>Point 1 0.000000000 0.000000000 0.000000000<br>Point 2 0.000000000 0.000000000 1.000000000
